# Material stocks and embodied carbon in UK buildings: An archetype-based, bottom-up, GIS approach

**DOI:** 10.1111/jiec.70066

**Published:** 2025-07-28

**Authors:** Charles Gillott, Maud Lanau, Elen Mitchell Reid, Farhana Sharmin, Danielle Densley Tingley

**Affiliations:** 1 Department of Civil and Structural Engineering, The University of Sheffield, Sir Frederick Mappin Building (Broad Lane Building), Mappin Street, S1 3JD Sheffield, UK; 2 Architecture and Civil Engineering, Chalmers University of Technology, Gothenburg, Sweden

**Keywords:** archetype, bottom-up, buildings, embodied carbon, industrial ecology, material stocks

## Abstract

Decarbonizing construction through a circular economy requires an in-depth understanding of the materials stocked within, and flows into, between, and beyond, the global built environment. Archetype-based bottom-up material stock analysis (MSA) is increasingly used to estimate the quantity, location, and embodied carbon of different construction materials within existing buildings. European MSA studies typically employ archetypes based on building use (e.g., residential/non-residential) and/or age (e.g., historic/modern), potentially missing significant variation in material composition across different construction types. Such work also generally focuses on residential buildings and derives aggregated and/or synthetic material intensities (MIs), with non-residential buildings in the United Kingdom rarely being considered through real-world design data. This paper investigates the suitability of different archetyping approaches in the bottom-up estimation of material stocks and embodied carbon in UK buildings. Concrete is revealed to consistently contribute the majority of material mass irrespective of use or construction type, with steel consistently representing a majority of superstructural embodied carbon. Despite the relative agreement between overall structural MIs for use- and construction-based archetypes, varying material and sub-/superstructural composition introduces increased heterogeneity in individual-material and sub-building MIs when considering building construction type. Mapping of use- and construction-based MIs to the building stock level is shown to be inhibited by the infrequent specification of construction type within the utilized inventory. This gives rise to a novel, parallel use and/or construction archetyping approach, reiterating the need for the inclusion of building construction types within both MSA archetyping approaches and national building inventories.

**Supplementary Information:**

The online version of this article (doi:10.1111/jiec.70066) contains supplementary material, which is available to authorized users.

## INTRODUCTION

The construction and use of buildings account for 43% of global greenhouse gas (GHG) emissions (United Nations Environment Programme, 2022), making them critical to limiting global average temperature increase and meeting decarbonization targets (United Nations Framework Convention on Climate Change, 2015). In the United Kingdom, around a quarter of emissions from buildings are in the form of embodied carbon (UK Green Building Council, 2021), resulting from the extraction, manufacture, and transport of materials; construction, maintenance, and demolition of buildings; and processing and disposal of waste (British Standards Institution, 2012). As operational building emissions continue to decrease, embodied carbon is expected to represent a majority of whole-life emissions by 2040 and almost three-quarters by 2050 (UK Green Building Council, 2021).

The growing pertinence of embodied carbon and concerns regarding resource scarcity and construction and demolition waste have led to increasing recognition of the need for a circular economy within the built environment. A circular economy (CE) minimizes material extraction, waste generation, and associated embodied carbon emissions by narrowing, slowing, and closing resource flows through efficient design, lifespan extension, and reuse and recycling (Bocken et al., 2016). In the case of buildings, this includes optimization of structural systems and design for longevity, adaptability, and deconstruct-ability; retention and reuse of existing assets; and prioritization of reused/reusable components and recycled/recyclable materials (Gillott et al., 2023).

With the mounting impetus for a CE, the increasing research focus is placed on the characterization of material stocks within and flows into, between, and beyond the global built environment (Lanau et al., 2019). “Bottom-up” material stock analysis (MSA) estimates total stocks by multiplying material intensities (MIs) of different assets by their corresponding size and/or number. This is typically more data- and labor-intensive than the “top-down” approach, requiring both detailed asset designs and a comprehensive asset inventory, but generally yields greater material disaggregation and spatial resolution (Lanau et al., 2019; Wuyts et al., 2022).

Following Tanikawa and Hashimoto's 4d-GIS (four-dimensional Geographic Information System) method in 2009, a growing number of bottom-up MSA studies have employed archetype-based approaches (Lanau et al., 2019; Wuyts et al., 2022). Adapted from the context of energy performance modeling, these represent heterogeneous building inventories as a series of homogeneous archetypes, enabling a suite of characteristic MIs to be calculated and extrapolated across the inventory. In the 4d-GIS approach, and typical across Chinese, Japanese, and North American studies, these archetypes are based upon building use (a.k.a. “typology” or “type,” e.g., “residential” or “non-residential”) and/or construction type (e.g., “traditional wooden house” or “reinforced concrete building”) (Arceo et al., 2021; Cheng et al., 2018; Hashimoto et al., 2009; Rankin et al., 2024; Tanikawa et al., 2014, 2015; Wang et al., 2015). As construction type is rarely detailed in European building inventories, building age (a.k.a. “cohort”) is often used as a proxy (Lanau & Liu, 2020; Ortlepp et al., 2016, 2018). This assumes a correlation between building age and construction type, shown to be only moderately true for the Chinese building stock (Zhang et al., 2022), potentially introducing significant uncertainty. Recognizing regional variation in archetyping approaches and associated MIs, the RASMI dataset synthesizes MIs for 32 regions across the globe, categorizing across three use (non-residential, multi-family residential, and single-family residential) and four construction (reinforced concrete, masonry, timber, and steel) archetypes (Fishman et al., 2024).

Bottom-up material stock studies generally focus on residential buildings, with non-residential buildings often being omitted (Arceo et al., 2021; Han & Xiang, 2013; Mastrucci et al., 2017; Mesta et al., 2019; Oezdemir et al., 2017; Ortlepp et al., 2018; Rankin et al., 2024; Schandl et al., 2020) or represented by a smaller number (i.e., 1–3) of archetypes (Fishman et al., 2024; Haberl et al., 2021; Lanau & Liu, 2020; Lanau et al., 2021; Miatto et al., 2019; Tanikawa & Hashimoto, 2009). The increased prevalence of residential buildings is because of the comparatively limited availability of non-residential design data, resulting from heightened commercial and political sensitivity and more diverse functions, structural forms, and materials (Lanau et al., 2019; Ortlepp et al., 2016). Despite being used to justify their limited consideration, the heterogeneity of non-residential buildings increases the need for their investigation as part of bottom-up MSA studies. This is furthered by their composition of high-value materials of varying reusability (e.g., steel and concrete) that, owing to typically shorter building lifespans (Lanau et al., 2019; Schebek et al., 2017), are more likely to become available as a secondary resource.

Despite consideration in numerous locations across Europe, Australia, Asia, and North/South America (Lanau et al., 2019; Wuyts et al., 2022), the bottom-up estimation of UK material stocks remains comparatively scarce (Wiedenhofer et al., 2024). Rather than accumulated stock, UK studies generally focus on associated flows (Ajayebi et al., 2021; Drewniok et al., 2023), consider only a single construction material (e.g., Davis et al., 2007; Geyer et al., 2007; Shanks et al., 2019), and/or derive synthetic MIs from hypothetical building designs (Ajayebi et al., 2021; Drewniok et al., 2023; Wiedenhofer et al., 2024). Such studies also characterize materials into broad groups (e.g., “steel”), overlooking significant variation in the embodied carbon secondary use potential of different material subgroups (e.g., “steel beams” vs. “steel reinforcement”).

To address concerns surrounding the use of age-based archetypes and aggregated synthetic MIs, this paper investigates the suitability of alternative archetyping approaches in the bottom-up estimation of material stocks and embodied carbon in UK buildings. In doing so, the following contributions to literature are made:

1. Quantification of the structural material and embodied carbon content of 30 UK buildings of different (non-)residential use and construction types.
2. Generation of a suite of building layer- and material-disaggregated MI and carbon intensity (CI) coefficients using different archetyping approaches.
3. Estimation of material stocks and embodied carbon at the building stock level using use- and construction-based archetyping.

## METHODS

**FIGURE 1:**
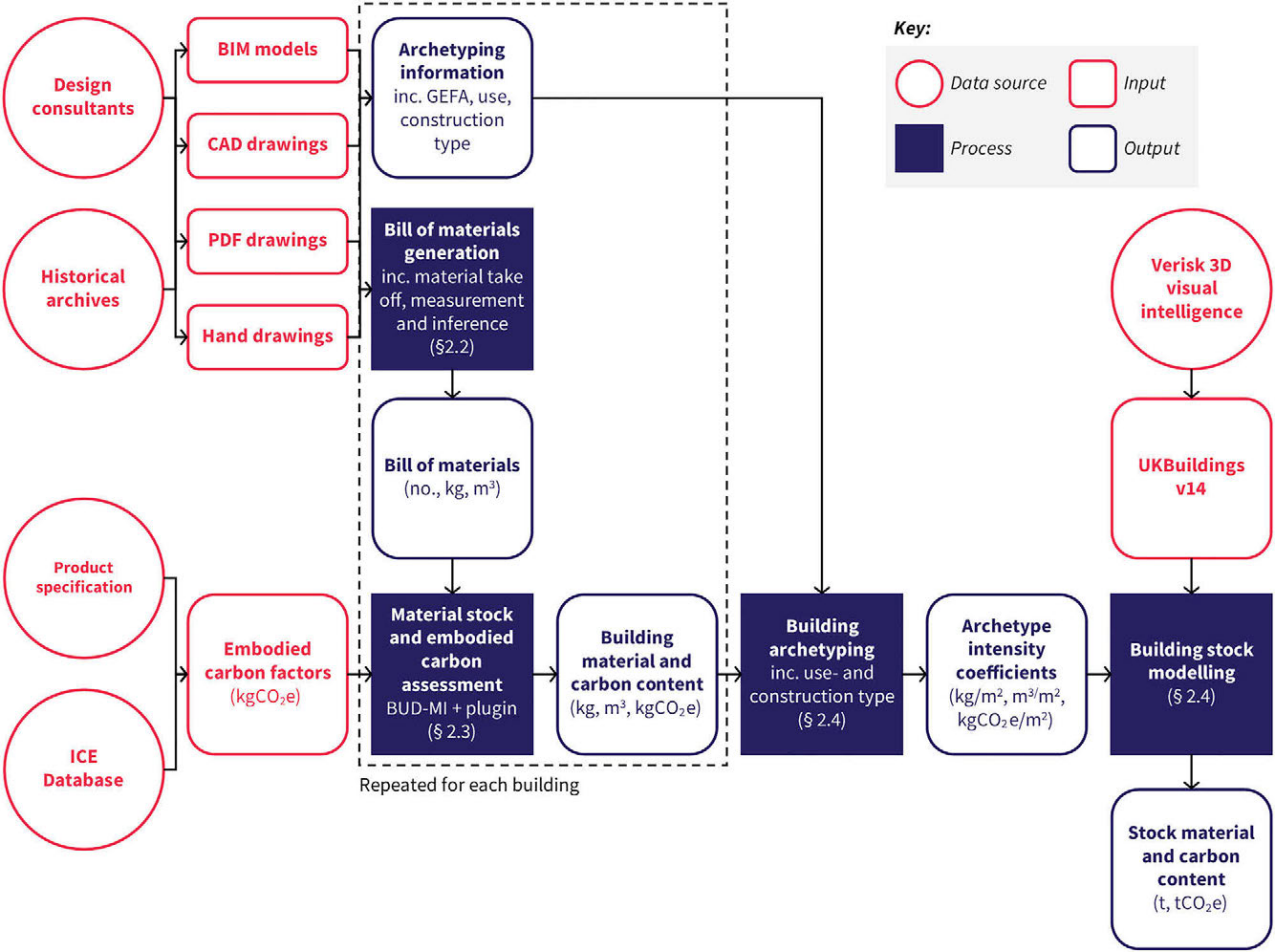
Overview of the developed methodology detailing data sources, inputs, outputs, processes, and associated manuscript sections. BIM, building information modeling; CAD, computer-aided design; PDF, portable document format; BUD-MI, bottom-up data: material intensity.

In line with existing work (Lanau et al., 2019; Wuyts et al., 2022), the developed methodological framework (Figure 1) follows an archetype-based, bottom-up, GIS approach. In this, following data collection (Section 2.1) and pre-processing (Section 2.2), the super- and sub-structural MIs of a suite of case study buildings are derived from architectural and engineering drawings using the BUD-MI (bottom-up data: material intensity) template (Lanau et al., 2024) (Section 2.3). This includes all load-bearing elements of each building's structure layer, as defined by Brand (1994). A UK-specific embodied carbon plug-in is also integrated (Figure 1) to calculate associated product stage (i.e., A1-A3) GHG emissions. Next, case study buildings are grouped into two discrete sets of archetypes, based on their use and construction type and representative material/carbon intensities M/CIs calculated (Section 2.4). Finally, the “UKBuildings” GIS building inventory (Verisk 3D Visual Intelligence, 2023) is used to extrapolate M/CIs to the building stock level, facilitating comparison of material stock and carbon emission estimations employing alternative archetyping approaches (Section 2.4).

### Building sampling

Architectural and engineering design data has been collected from design consultancies and historical building archives. Initially, design information was obtained for 48 buildings, including digital BIM (building information modeling, *n* = 33), CAD (computer-aided design, *n* = 8), and PDF (portable document format, *n* = 9) files, as well as physical printouts and hand-drawn records (*n* = 5). Of these 48 buildings, 18 were excluded from analysis as a result of insufficient/incomplete data and/or misalignment with project scope (e.g., omitting entire building areas or detailing only non-structural elements) to result in a final sample of 30.

### Bill of materials generation

Bills of materials for each building were generated employing a range of approaches for different data formats. For BIM models, the “material takeoff” function in Autodesk Revit was used to generate a schedule of building components, detailing their material composition, location within the building, volume/mass, and quantity. For buildings modeled in CAD, a comparable bill of materials was derived through manual inference of design drawings and the measurement of dimensions and quantities in AutoCAD. A similar process was followed for PDF and physical drawings, with component locations, materials, volumes/masses, and quantities being parsed from existing labels and annotations. Following generation, a formal check of each bill of materials was conducted by an independent member of the research team (i.e., someone not involved in its generation) to ensure its alignment with raw design data.

### Material stock and embodied carbon assessment (BUD-MI)

For each building in turn, the key bill of materials data (e.g., item location, composition, and volume/mass) was imported into BUD-MI, an Excel-based data template for the harmonized collection of material intensity data (Lanau et al., 2024). This saw manual specification of each item's building layer (e.g., “structure” or “skin”) (Brand, 1994) and element (e.g., floor or wall), as well as its location above or below ground. Material composition was detailed using a four-tiered system in which material groups (e.g., metals) were sequentially refined into materials (e.g., steel) and then material sub-groups (e.g., steel reinforcement) through a series of nested lists. This facilitated auto-population of material properties (e.g., density) predefined within BUD-MI, as well as hierarchical aggregation within material types for data reporting purposes (Section 3.2). Items with solely dimensional data were converted to associated masses using these predefined densities, with informed assumptions on material grade/specification being made and recorded in BUD-MI where not explicitly detailed.

A UK-specific embodied carbon plug-in was also integrated within BUD-MI, auto-populating embodied carbon factors from the Inventory of Carbon and Energy (i.e., ICE database) (Jones & Hammond, 2019) and relevant environmental product declarations. As in previous work (e.g., Lanau et al., 2021), this approximates product stage (i.e., A1-A3, “cradle-to-gate”) (British Standards Institution, 2012) global warming potential in terms of carbon dioxide equivalents (CO_2_e), with UK-specific values being used in place of European/global averages where available. This process followed Royal Institution of Chartered Surveyors guidance throughout (e.g., for the sequestration of biogenic carbon), representing best practice in the UK context (RICS, 2021). Building-level information required for the calculation of material and carbon intensities (Section 2.4) and exploration of archetyping approaches (Section 2.4) was also recorded in BUD-MI, including use, construction type, and gross external floor area (GEFA) (RICS, 2021).

Following verification by an independent member of the research team (i.e., someone not involved in its generation), the relevant data within each BUD-MI template (inc., building use, construction type, and GEFA and material mass, volume, and embodied carbon) was outputted as a CSV (comma separated value) file. This enabled the subsequent calculation of M/CIs for each building by dividing material and carbon mass by GEFA.

### Building archetyping and stock modeling

To facilitate the investigation of different archetyping approaches, each of the 30 buildings was assigned both a use and construction archetype using the definitions in Table 1. From this, representative M/CIs were calculated as the mean of the individual building values for a given archetype. Owing to the non-representativeness of building ages within the considered sample, investigation of age-based archetypes was not considered herein.

**TABLE 1:** Construction- and use-based archetype definitions and considered sample size.

Archetyping approach	Archetype name	Archetype definition	Sample size (number of buildings)
Construction-based	Steel frame	Buildings for which the majority floor/roof area is supported primarily by a steel superstructure, comprising column and beam elements.	12
	Concrete frame	Buildings for which the majority floor/roof area is supported primarily by a concrete superstructure comprising precast and/or in situ columns/walls and beams/floors.	13
	Timber frame	Buildings for which the majority of floor/roof area is supported primarily by a timber superstructure comprising timber and/or mass timber columns/walls and beams/floors.	5
Use-based	Office	Buildings intended for use as “an office to carry out any operational or administrative functions” (The Town & Country Planning (Use Classes) Order 1987, 2023).	9
	Educational	Buildings intended for “the provision of education” (The Town and Country Planning (Use Classes) Order 1987, 2023).	8
	Industrial	Buildings intended for “any industrial process […] [including] storage or as a distribution centre […] incineration purposes, chemical treatment, landfill or hazardous waste” (The Town and Country Planning (Use Classes) Order 1987, 2023).	4
	Apartment	Buildings with subdivided units intended “for the use as a dwellinghouse by a single person or by people to be regarded as forming a single household” (The Town and Country Planning (Use Classes) Order 1987, 2023).	6
	Retail	Buildings intended “for the display or retail sale of goods, other than hot food, principally to visiting members of the public” (The Town and Country Planning (Use Classes) Order 1987, 2023).	1
	Religious	Buildings intended “for, or in connection with, public worship or religious instruction” (The Town and Country Planning (Use Classes) Order 1987, 2023).	1
	Recreational	Buildings intended “for the principal use of the local community” and “visiting members of the public” including “a hall or meeting place’ or ‘for indoor sport, recreation or fitness” (The Town and Country Planning (Use Classes) Order 1987, 2023).	1

Following their calculation, archetype M/CIs were mapped to individual buildings within the UKBuildings inventory using specified use and construction type attributes and the definitions in Table 1. UKBuildings is a proprietary GIS database from Verisk Analytics, detailing building geometry (e.g., footprint and height) and characteristics (e.g., age, use, and construction type) across England, Scotland, and Wales and Northern Ireland (Verisk 3D Visual Intelligence, 2023). Owing to the frequent omission of use or construction type attributes in UKBuildings and consideration of a subset of UK archetypes herein (Section 3.4), only a portion of the UK inventory was able to be modeled through this process. To mitigate the effects of this and further explore the suitability of alternative archetypes, a parallel archetyping approach was also adopted. In contrast to the application of use or construction archetyping *across the inventory as a whole*, this saw use- or construction-based M/CIs assigned on a *building-by-building basis*. Inclusion of both use and construction type for some buildings within the inventory gives rise to two variants of this parallel approach, prioritizing use- or construction-based archetyping respectively (see Figure 5). An additional analysis of this subset of buildings to which a use *and* construction archetype pertain has also been considered, facilitating a comparison between use and construction archetyping at the building stock level.

## RESULTS AND DISCUSSION

### Building sample

Of the 30 buildings analyzed (Section 2.1), 13 (43%) were concrete framed, 12 (40%) steel framed, and 5 (17%) timber framed (Table 1). Considering respective historic non-residential market shares of 25%, 65%, and 3% (British Constructional Steelwork Association, 2021) and the increased prevalence of concrete frames in multi-unit residential buildings, this is thought to be broadly proportional to the composition of the United Kingdom's mid-high rise building stock. The analyzed sample was distributed across a range of different uses, including 9 (30%) office, 8 (27%) educational, 4 (13%) industrial, and 6 (20%) apartment, as well as single buildings (3% respectively) of retail, religious, and recreational use.

### Material and carbon intensity

#### Building material intensity

**FIGURE 2:**
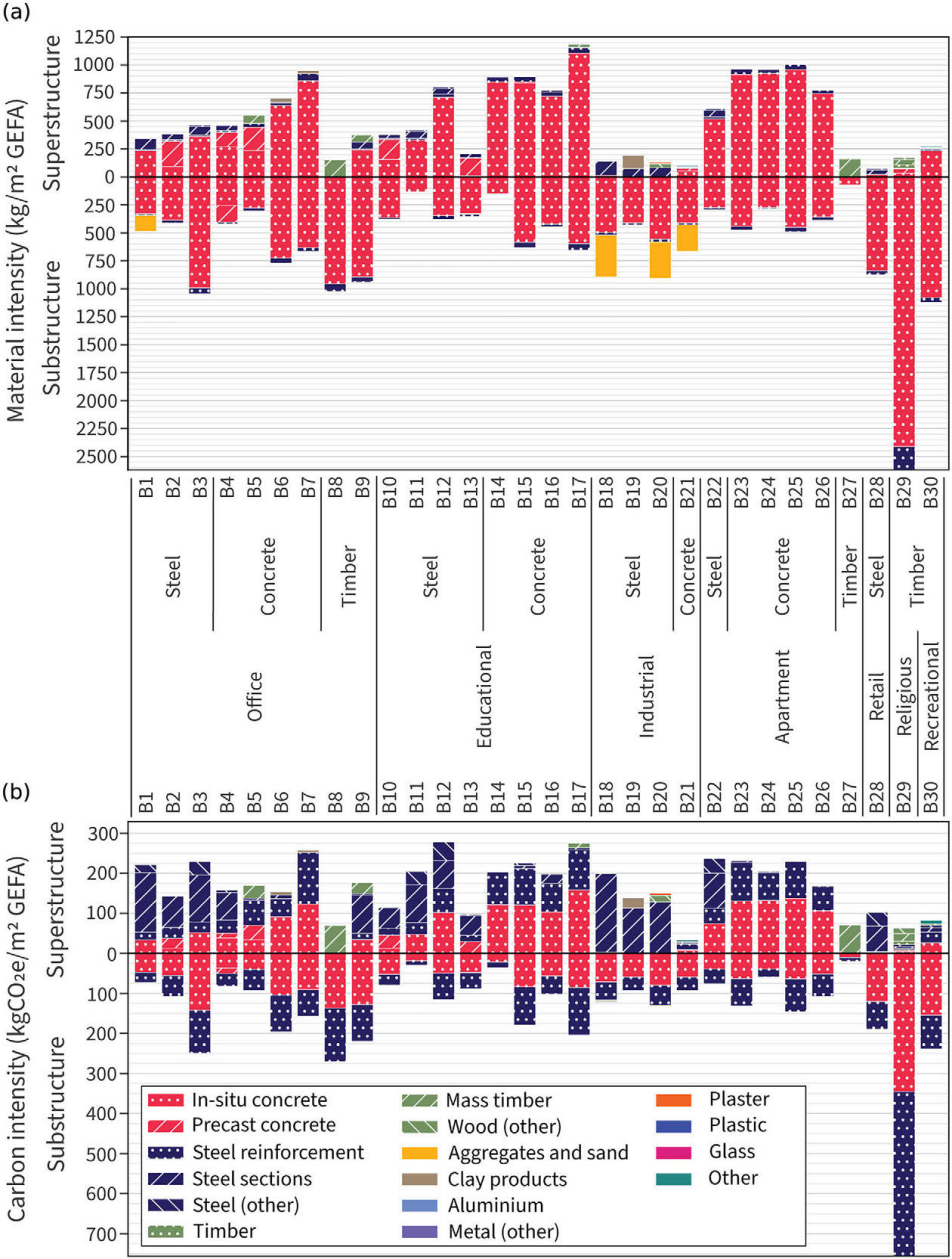
Mass (kg/m^2^) (a) and carbon (kgCO_2_e/m^2^) (b) intensity of different materials in the super- and substructural layers of the 30 buildings (B1–B30) analyzed (underlying data for this figure are provided in Tables 1 and 2 of Supporting Information S1).

Figure 2 shows material-disaggregated M/CI for each building's sub- (i.e., below ground) and super- (i.e., above-ground) structural layers. Overall MIs vary by a factor of more than 10 across the considered buildings, ranging from 238 to 2791 kg/m^2^ for B27 (timber-apartment) and B29 (timber-religious), respectively (Figure 2a). Accounting for variations in assessment scope (i.e., considered location, building layers, materials, and archetypes), the overall MIs in Figure 2a are broadly in line with those from previous studies (Drewniok et al., 2023; Tanikawa & Hashimoto, 2009; De Wolf et al., 2016). One exception to this is the work of Ortlepp et al. (2016, 2018) in which MIs are around twice those in Figure 2a (e.g., averaging 2600 kg/m^2^ for “office and administrative” uses, 2500 kg/m^2^ for “factory and workshop,” and 2124–2815 kg/m^2^ for different types of “multi-family house”). Considering Ortlepp et al.’s derivation of non-synthetic MIs from real-world design data, as in this study, the most likely causes of this disparity are international variation in construction practices and/or the inclusion of additional (i.e., non-structural) building elements within their work. This highlights the need for clarity and consistency in considered building extents, substantiating the move to a layer-disaggregated approach as presented herein.

The amount of material associated with sub- and superstructural elements is variable across the sample, as well as proportionally within each building. This is observed most dramatically between B29 (timber-religious), which has the greatest substructural MI (2619 kg/m^2^) despite one of the smallest superstructural MIs (172 kg/m^2^), and B6 (concrete-office), which has almost identical super- and substructural intensities of 703 and 772 kg/m^2^, respectively (Figure 2a). Such large disparity may be attributed to variations in the presence of habitable substructural space (e.g., basements), local ground conditions, and superstructural loading. For example, despite having no basement, B29 has a disproportionately large substructural mass in order to resist significant uplift forces on the building's superstructure.

With the exception of B27 (timber apartment), and irrespective of use and construction type, Figure 2a reveals the primary material within each building to be concrete. In non-concrete framed buildings, alongside in situ and precast flooring, this is largely attributed to in situ concrete substructures (e.g., basements and foundations), which often outweigh superstructural materials (Figure 2a). Partially because of this in situ concrete content, the second most prevalent material within the majority of buildings is steel, with reinforcement occurring in rough proportionality to concrete in both super- and substructural components. Beyond this, steel sections make up an additional 31 kg/m^2^ (B13) to 126 kg/m^2^ (B18) in steel-framed buildings, with other steelwork (e.g., fabricated components and composite floor decking) contributing up to 17 kg/m^2^ (B12). This sees steel contribute just 11% (B13) to 14% (B18) of total material mass in steel-framed buildings.

#### Archetype material intensity

**FIGURE 3:**
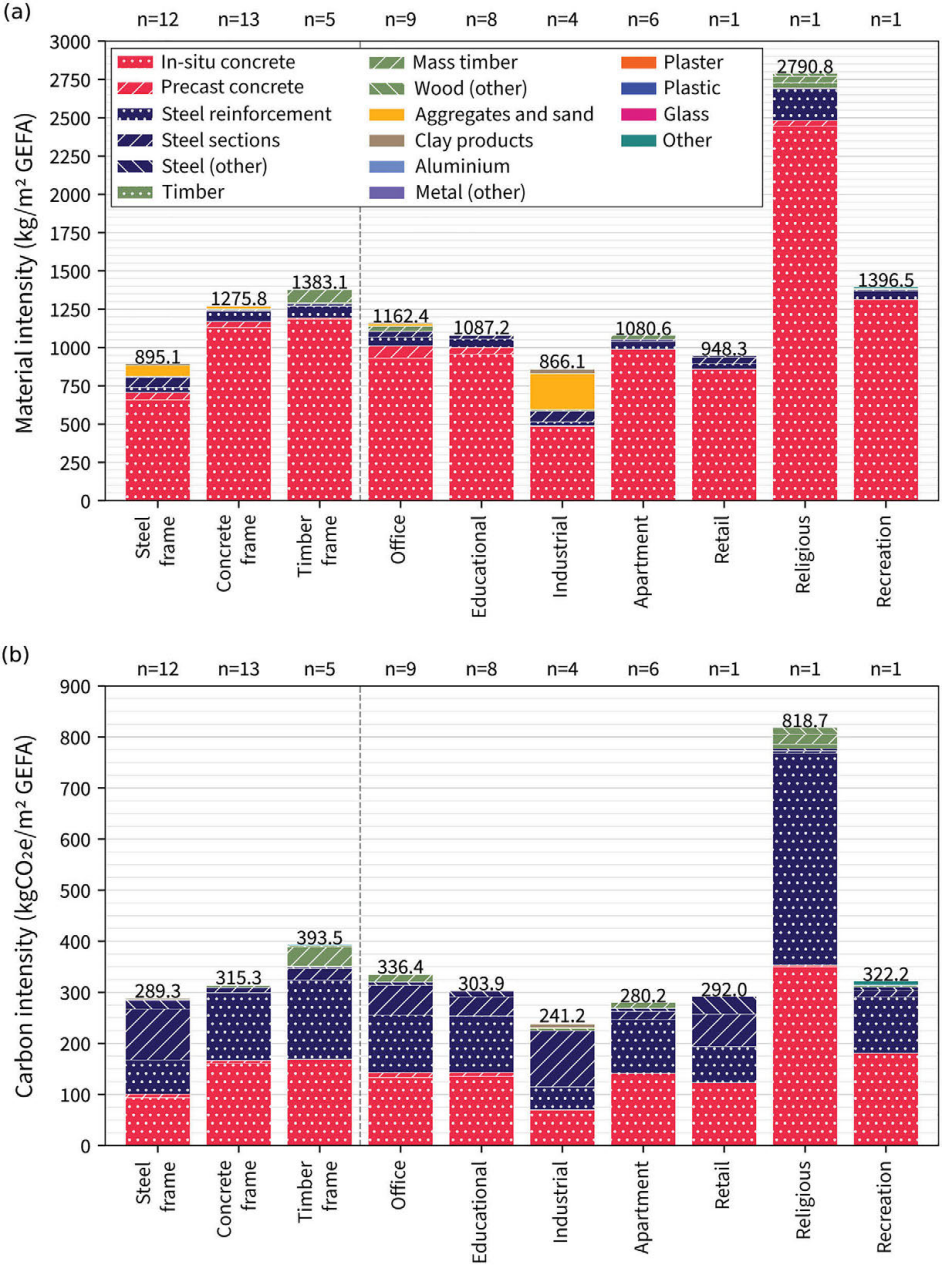
Mass (kg/m^2^) (a) and carbon (kgCO_2_e/m^2^) (b) intensity of different materials within the structural layer of the 30 buildings (B1–B30) analyzed (underlying data for this figure are provided in Tables 1 and 2 of Supporting Information S1).

Figure 3a shows the structural layer of concrete-framed buildings to be more materially intense than those with steel frames, with average values of 1276 and 895 kg/m^2^, respectively. Considering their similar substructural MIs (489 and 550 kg/m^2^ for concrete and steel on average), this is largely due to their contrasting superstructural composition, with the average above-ground mass of concrete and steel framed buildings (786 and 345 kg/m^2^, respectively) being broadly in line with previous estimations for non-residential and multi-family buildings in Europe (Fishman et al., 2024). Despite having less materially intense superstructures (226 kg/m^2^ on average), timber-framed buildings generally exhibit greater substructural MIs (1157 kg/m^2^ on average) than those with steel or concrete frames (Figure 3a). This is heavily influenced by the potentially anomalous substructural material intensity of 2619 kg/m^2^ for B29 (timber-religious), however, excluding which the average substructural MI reduces to 792 kg/m^2^.

Although variables between individual buildings, office, educational, and apartment buildings have similar MIs on average, with values of 1162, 1087, and 1081 kg/m^2^, respectively. These values are in general agreement with existing studies, though represent a relative increase and decrease compared with the work of De Wolf et al. (2016) and Drewniok et al. (2023) and exhibit greater similarity between residential and non-residential buildings than is typically seen. Potential reasons for this disparity include Drewniok et al.’s consideration of complete-yet-hypothetical building designs and the greater prevalence of concrete framed (and thus more materially intense) apartments and taller (and thus less materially intense) office buildings in the sample considered herein. In closer agreement with Drewniok et al. (2023), Figure 3a shows industrial buildings to exhibit much lower MIs overall, averaging just 866 kg/m^2^. This is because they are typically only single-story structures, with the sample's frequent use of steel portal frames being more materially efficient than the multi-story construction types employed in other uses. Superstructural elements contribute just 141 kg/m^2^ of the overall material content of industrial buildings on average, with the remaining 725 kg/m^2^ resulting from the buildings’ substructure. Because of the absence of basements in all industrial buildings considered, this substructural intensity is likely to result from enhanced ground floor slabs and the associated foundations often required by industrial activities.

#### Carbon intensity

Overall embodied carbon intensity varies by a factor of 9 across the considered buildings, ranging from 90 to 819 kgCO_2_e/m^2^ for buildings B27 (timber-apartment) and B29 (timber-religious), respectively (Figure 3). Owing to variation in embodied carbon factors, generally taken as 1.55 and 0.143 kgCO_2_e/kg for steel and concrete, respectively (Jones & Hammond, 2019), and despite concrete's dominance by mass (Figure 2a), steel components make a majority contribution to overall carbon intensity in the majority of buildings (Figure 2b). Similarly, although most of a building's mass is typically substructural (Figure 2a), the majority of associated embodied carbon can be found within superstructural elements (Figure 2b). This is particularly interesting when considering the circular economic potential of building material stocks, with above-ground elements having inherently higher recoverability than those buried within a building's substructure. Behind steel, concrete is generally the second largest contributor of embodied carbon across the considered buildings (Figure 2b). Owing to its prevalence within foundations, composite floors, and concrete frames, this is most often as in situ concrete, but also as precast floor panels in some steel-framed buildings.

Despite comprising varying proportions of different construction types (Figure 2), the average embodied carbon intensity of office (336 kgCO_2_e/m^2^), educational (304 kgCO_2_e/m2), apartment (290 kgCO_2_e/m2), and industrial (241 kgCO_2_e/m2) buildings follows the same rank order as for material mass (Figure 3b). In further similarity, and again because of variations in assessment scope (e.g., Drewniok et al's consideration of lifecycle stages A4–A5), these values are, respectively, higher and lower than those obtained by De Wolf et al. and Drewniok et al. A greater similarity is seen with the work of Simonen et al. (2017), especially in the case of office (399 kgCO_2_e/m^2^) and educational (385 kgCO_2_e/m^2^) buildings, despite this work's variable assessment scope (i.e., considered buildings layers and lifecycle stages).

### Archetyping approaches

**FIGURE 4:**
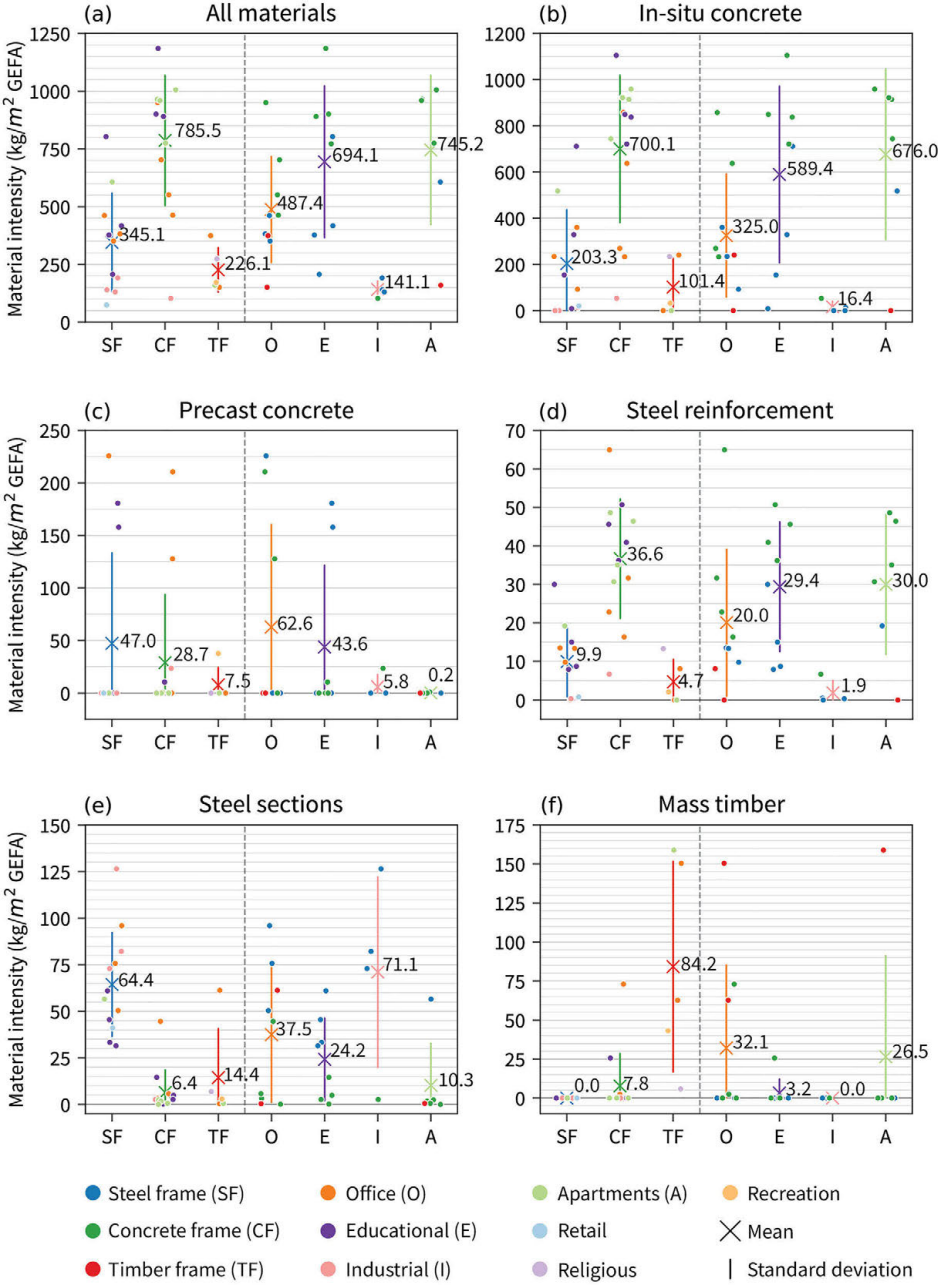
Superstructural intensity (kg/m^2^) of all materials (a), in situ concrete (b), precast concrete (c), steel reinforcement (d), steel sections (e), and mass timber (f) in buildings of different construction and use archetype (underlying data for this figure are provided in Table 1 of Supporting Information S1).

Although contributing a significant and varying proportion of structural material mass, building substructures are heavily dominated by reinforced concrete and thus more compositionally consistent than building superstructures (Figure 2). This is particularly true when considering material sub-groups, with in situ concrete and associated steel reinforcement representing a majority contribution to substructural mass and embodied carbon across all buildings and associated archetypes (Figure 2). For this reason, and the typically increased embodied carbon content (Figure 2) and secondary use potential of superstructural materials, exploration of the suitability of different archetyping approaches considers only building superstructures. Figure 4 thus shows the superstructural mass intensity of the five most common material subgroups (i.e., in situ concrete, precast concrete, steel reinforcement, steel sections, and mass timber) in all buildings within each construction and use archetype. For completeness, a corresponding figure for building substructures is provided as Supporting information S2, reiterating the comparatively limited compositional variation in material content when compared with building superstructures.

As in Section 3.2, Figure 4a shows the average superstructural MI of concrete-framed buildings to be more than twice that of steel-framed buildings and more than three times those with timber frames. Apartment buildings are revealed to have the most materially intense superstructures overall, with an average value of 745 kg/m^2^ across the archetype subsample of 6. This contrasts with the findings of previous work (Drewniok et al., 2023) as well as what would be expected when considering the lower imposed loads associated with residential buildings in the United Kingdom (British Standards Institution, 2002). As suggested in Section 3.2, the heightened MI of apartment buildings is revealed by Figure 4a to result from the inclusion of a larger proportion of concrete framed buildings than is seen for other use types. Following apartments, educational (694 kg/m^2^) and office (487 kg/m^2^) buildings have sequentially less materially intense superstructures on average, with Industrial buildings having the lowest average material content of just 141 kg/m^2^.

In part because of this low average value, industrial buildings exhibit the greatest internal consistency of all use archetypes, with the same being true for timber framed buildings when compared with other construction types (Figure 4a). Aside from these examples, Figure 4a shows large variation from the mean across all archetypes and thus no clear preference for those that are use- or construction-based. Notwithstanding this, when considering the use archetypes in Figure 4a, there is a clear divergence of superstructural MIs for buildings of different construction types, with concrete-framed buildings typically distributed above the mean archetype value and steel- and timber-framed buildings below this (Figure 4a). This indicates the potential for consideration of construction type in bottom-up material stock studies to generate more homogeneous building archetypes than those based solely on use.

With the exception of industrial buildings, and similarly, as for building super- and sub-structures in Section 3.2, Figure 4 shows concrete to be the primary contributor to average superstructural MI across all archetypes. By and large, this is due to the prevalence of in situ concrete, with contributions from precast units generally being smaller by an order of magnitude (Figure 4). Concrete's dominance is perhaps most interesting in the case of steel- and timber-framed superstructures, which, on average, contain greater amounts of in situ concrete than do steel sections and mass timber, respectively. When considering the use archetypes in Figure 4b,d,e, there is a clear distinction between different construction types, with in situ concrete and steel reinforcement typically occurring in above-average quantities in concrete-framed buildings and steel sections typically occurring in above-average quantities in steel-framed buildings. Though unsurprising, such nuance is not accounted for when applying solely use archetypes, reiterating the importance of considering building construction type in future MSA studies. This is similarly true for the alternating prevalence of in situ and precast concrete in steel and concrete buildings (Figure 4b,c), suggesting the potential to further disaggregate these archetypes going forward.

Alongside material stock mass, variations in superstructural composition identified through consideration of construction-based archetypes and disaggregated material subgroups begin to highlight implications for associated embodied carbon and secondary use potential. This is true for precast (0.249 kgCO_2_e/kg) and in situ (0.143 kgCO_2_e/kg) concrete elements (Jones & Hammond, 2019), with the latter having a significantly reduced reuse potential despite its lower upfront embodied carbon as a result of its reliance on non-reversible chemical connections (Gillott et al., 2023). In the case of steel sections (1.55 kgCO_2_e/kg) and reinforcement (1.99 kgCO_2_e/kg) (Jones & Hammond, 2019), lower embodied carbon is aligned with increased circularity, with ease of recovery meaning the former is typically suitable for elemental reuse and the latter limited to recycling (Gillott et al., 2023). Such considerations of circular economy potential are highly dependent on a number of context-specific architectural and engineering factors (e.g., adaptability, durability, and recoverability), however, and should be explored as part of future work.

### Building stock modeling

**FIGURE 5:**
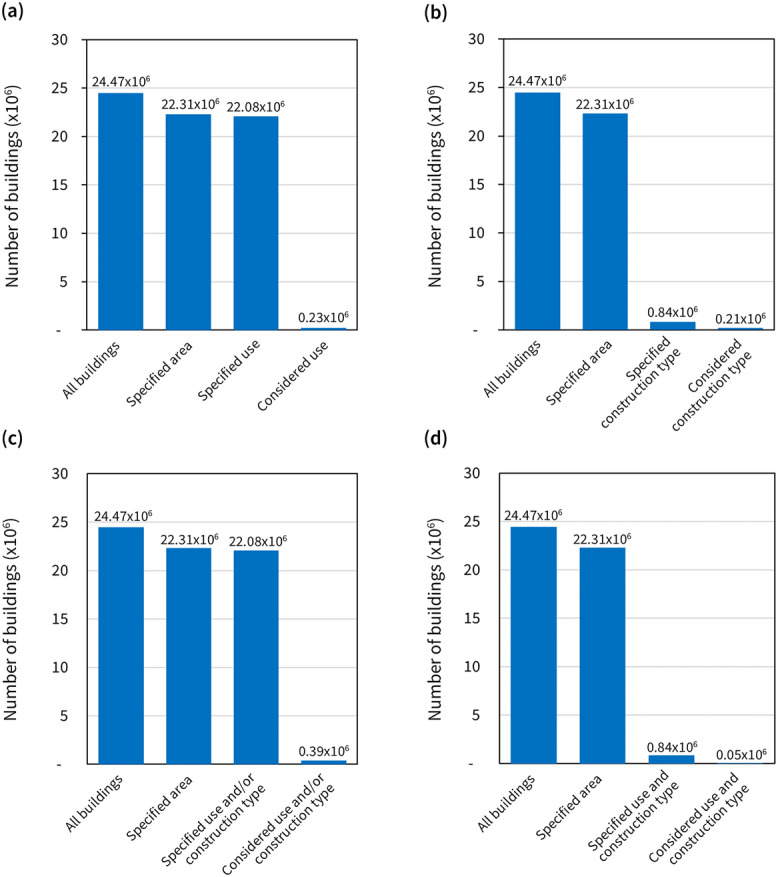
Number of buildings within the UKBuildings inventory of specified floor area (a–d), use (a), and construction type (b), and within a considered use (a), construction type (b), use and/or construction type (c), and use and construction type (d) (underlying data for this figure are provided in Table 3 of Supporting Information S1).

When modeling material and carbon content at the building stock level (Section 2.43), the proportion of the UKBuildings inventory able to be considered reduces significantly in size as a result of the frequent non-specification of floor area, use, and construction type (Verisk 3D Visual Intelligence, 2023). Further reductions in the modeled inventory are seen when mapping specified uses (Figure 5a) and construction types (Figure 5b) to the archetypes considered herein (Section 2.4), with a parallel use and/or construction type archetype approach offering the potential to increase the number of buildings able to be modeled to 391,743 (Figure 5c).

**FIGURE 6:**
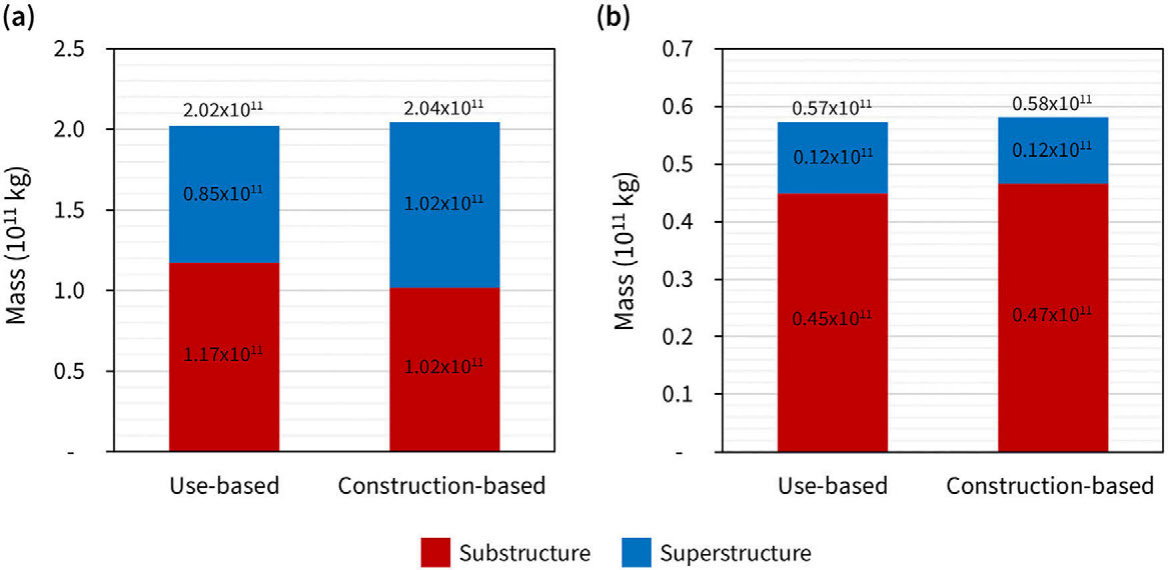
Sub- and superstructural material (a) and embodied carbon (b) mass of the 50,833 buildings of considered use and construction type, estimated using use and construction archetyping (underlying data for this figure are provided in Tables 4 and 5 of Supporting Information S1).

As shown in Figure 6, the total structural material mass of the 50,833 buildings of considered use *and* construction archetype varies by just 1% across alternate archetyping approaches. Along with a variation of 1.5% in associated embodied carbon (Figure 6b), this indicates relative consistency between use and construction archetypes when estimating whole-building material mass and embodied carbon at the building-stock level. An increased variation of 19% in superstructural material mass (Figure 6a) reveals this not to be the case when considering material stocks at the sub-building resolution, reiterating the need for increased inclusion of construction type in building stock modeling.

**FIGURE 7:**
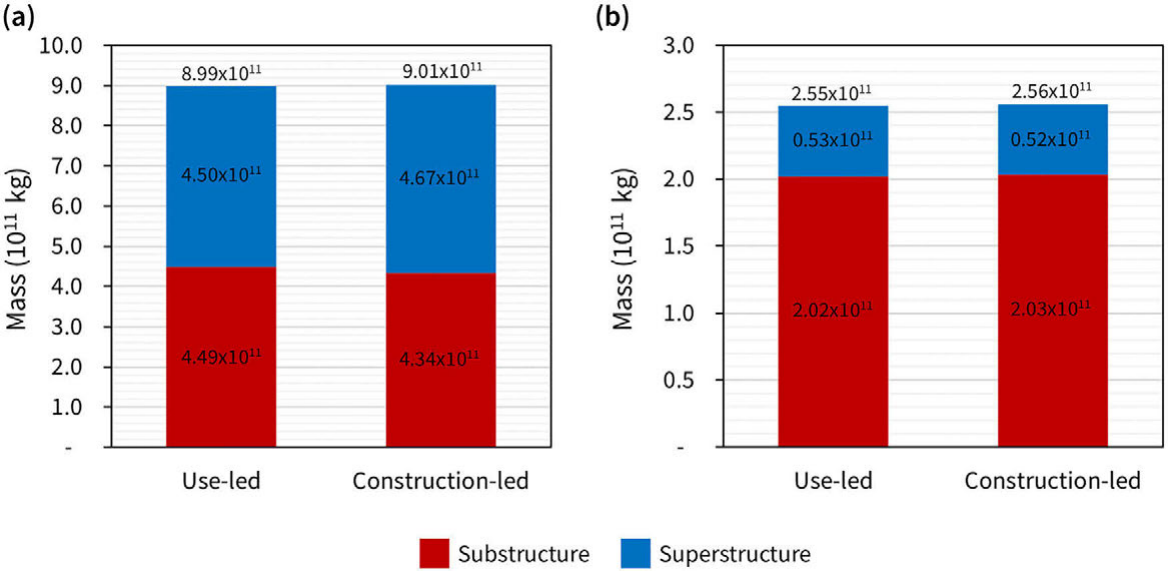
Sub- and superstructural material (a) and embodied carbon (b) mass of the 391,743 buildings of considered use and/or construction type, estimated using use- and construction-led parallel archetyping approaches (underlying data for this figure are provided in Tables 6 and 7 of Supporting Information S1).

Figure 7 reveals the total structural material content of the 391,743 buildings of considered use *and/or* construction type to vary by just 0.23% when employing use- and construction-led parallel archetyping approaches. The corresponding variation in embodied carbon is 0.34%, with similarly low variation between sub- and superstructural mass and embodied carbon.

Together, Figures 5–7 indicate no clear preference for use- or construction-based archetyping in the estimation of structural material and embodied carbon at the building stock level, but that a hybrid use and/or construction-type approach should be prioritized when utilizing sparsely attributed building inventories. In instances where intra-building (e.g., sub-/superstructural material mass/embodied carbon) or material-specific insights are required, the disparity in stock-level estimations (Figure 6) and the potential for increased heterogeneity in construction-based archetyping (Section 3.3) highlight a preference for construction-based and layer-disaggregated building archetypes.

### Limitations and future work

In meeting its aims and objectives (Section 1), this study presents a number of limitations with potential to be addressed in future work. As exemplified by the omission of 18 buildings from the already-small sample of 48 (Section 3.1), the poor availability and quality of design data are perhaps the most apparent of these. Although common within bottom-up MSA (Lanau et al., 2019; Ortlepp et al., 2016), associated limitations in gathering design data have dictated the use of a nonprobabilistic sampling approach herein and thus precluded the consideration of a statistically representative sample. This has resulted in a potential bias toward particular building ages, locations, and sizes.

As an alternative, a proportionate stratified random sampling approach would be of preference here, allowing buildings to be randomly selected to represent each archetype in proportion to their prevalence across the building stock. This process would require a comprehensive design repository for UK buildings and a more accurate and complete UK building inventory, the development of which is recommended for future work. Accessing design data in a consistent format such as this would also assist in reducing potential variation in the scope and detail of generated bills of materials across PDF/hand drawings and CAD/BIM models.

Enhanced inventory quality would also serve to mitigate issues associated with the limited coverage of archetype attributes observed within this work (Section 3.4) and enable geospatial estimation of material stocks and embodied carbon at the national scale. There is also potential to achieve both greater asset coverage and more homogeneous archetypes in future work, through consideration of additional use and construction types and further disaggregation of those considered herein. As suggested in Sections 3.3 and 3.4, this could see the inclusion of masonry buildings and single-unit housing as well as the separation of precast and in situ concrete frames and steel buildings with precast and in situ concrete floors. Further advancement of the project scope is also recommended, including the consideration of additional lifecycle stages (i.e., beyond A1–A3) and building layers (e.g., skin, space, and stuff).

## CONCLUSION

There are growing concerns regarding the employment of use- and age-based building archetypes and aggregated material classifications in bottom-up material stock analyzed. Previous UK studies also generally focus on residential buildings and/or derive synthetic MIs from hypothetical building designs. To address this, the presented work has investigated the suitability of different archetyping approaches in the bottom-up estimation of material stocks and embodied carbon in UK (non-)residential buildings. This includes quantification of the material and embodied carbon content of 30 case study buildings, generation of a suite of layer- and material-disaggregated material and carbon intensity coefficients, and the estimation of material stocks and embodied carbon at the UK stock level through the use, construction, and parallel archetyping approaches.

Concrete is the primary contributor to overall structural MI across the considered buildings, irrespective of their use and construction type. This is similarly true for both sub- and superstructural elements, with steel making a comparatively minimal contribution to superstructural MIs even in steel-framed buildings. Despite the dominance of concrete by mass, the higher per-unit carbon intensity of steel results in its majority contribution to superstructural embodied carbon across the buildings and archetypes considered.

Total structural MIs for the considered use and construction archetypes are relatively consistent, ranging from 866 to 1162 kg/m^2^ (for industrial and office buildings) and 895 to 1383 kg/m^2^ (for steel and timber frames), respectively. Total and individual superstructural MIs are more variable than this, with a typically greater prevalence of steel sections in industrial, office, and educational buildings and in situ concrete and associated reinforcement in apartments. Despite showing some correlation between use and construction types, clear divergence within use-based archetypes (e.g., concrete and steel framed buildings typically being distributed above and below the mean value, respectively) indicates the potential to increase the homogeneity of total superstructural M/CIs though the consideration of building construction type. Although unsurprising, this is furthered when considering individual material M/CIs within construction archetypes, with in situ concrete and steel reinforcement typically occurring in above-average quantities in concrete-framed buildings and steel sections typically occurring in above-average quantities in steel-framed buildings. Owing to the inherent disparity in the potential for secondary use of different material sub-groups (e.g., precast vs. in situ concrete and steel sections vs. reinforcement), such variation in material composition highlights associated implications for the circular economy potential of UK building archetypes. Further exploration of this is recommended as part of future work.

When modeling material and carbon content at the building-stock level, less than 1% of the UKBuildings inventory is able to be considered for both use- and construction-based archetyping. Besides, the exclusion of dominant UK archetypes (e.g., masonry structures and single unit housing) within this work is because of the frequent omission of construction type (97% of instances) within the UKBuildings inventory. Combined with the potential for increased M/CI homogeneity and exemplified increase in inventory coverage when employing a parallel archetype approach, this reiterates the need for increased consideration of building construction type within bottom-up MSA in the future.

## Supplementary Information

**Supporting Information 1**: Underlying data used for graphical figures.

**Supporting Information 2**: Supplemental figure of substructural material intensities for disaggregated material groups

## Data Availability

Research data are not shared.
