# Supplementary material for: Material stocks and embodied carbon in UK buildings: An archetype-based, bottom-up, GIS approach
Source: J Ind Ecol. 2025 Jul 28;29(5):1748–62. doi: 10.1111/jiec.70066 (PMC13275533; doi:10.1111/jiec.70066)
Supplement: Supplementary file 2 — Supporting Information 2: Supplemental figure of substructural material intensities for disaggregated material groups [file 44498_2025_2905019_MOESM2_ESM.docx]

**Supporting Information 2**: Supplemental figure of substructural material intensities for disaggregated material groups


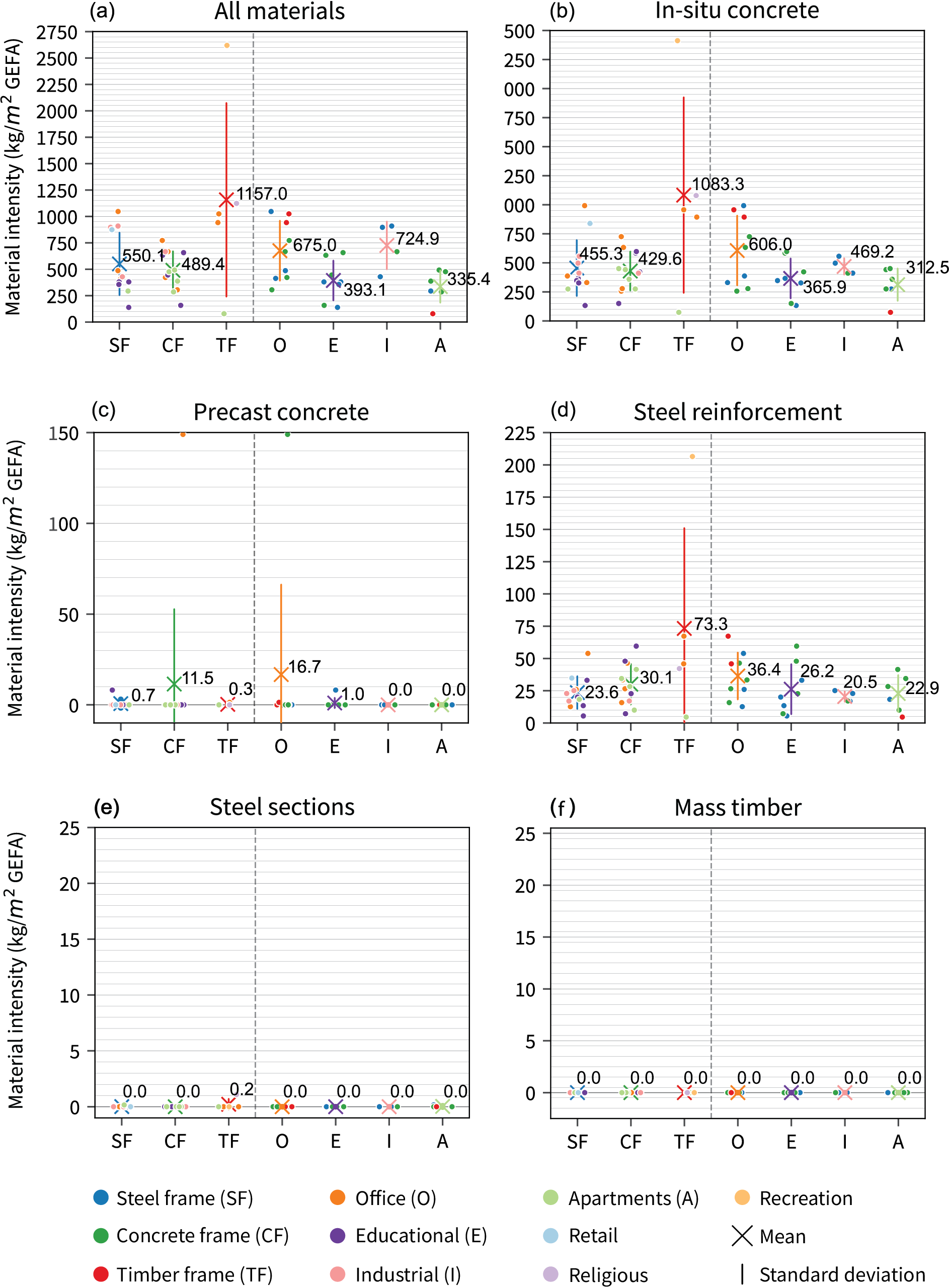


**Figure S**1 - Substructural intensity (kgCO2e/m2) of all materials (a), in-situ concrete (b), precast concrete (c), steel reinforcement (d), steel sections (e) and mass timber (f) in buildings of different construction and use archetype.
